# Characterization and expression profiling of the *ICE-CBF-COR* genes in wheat

**DOI:** 10.7717/peerj.8190

**Published:** 2019-11-29

**Authors:** Jie Guo, Yongkang Ren, Zhaohui Tang, Weiping Shi, Meixue Zhou

**Affiliations:** 1 College of Agronomy, Shanxi Agricultural University, Taigu, China; 2 Research Center of Biotechnology, Shanxi Academy of Agricultural Sciences, Taiyuan, China; 3 School of Land and Food, University of Tasmania, Hobart, Australia

**Keywords:** Expression patterns, *Triticum aestivum*, *ICE-CBF-COR*

## Abstract

Cold stress is one of the major abiotic stresses that limit crop production. The *ICE-CBF-COR* pathway is associated with cold stress response in a wide variety of crop species. However, the *ICE*-*CBF*-*COR* genes has not been well characterized in wheat (*Triticum aestivum*). This study identified, characterized and examined the expression profiles of the *ICE, CBF* and *COR* genes for cold defense in wheat. Five *ICE* (inducer of *CBF* expression) genes, 37 *CBF* (C-repeat binding factor) genes and 11 *COR* (cold-responsive or cold-regulated) genes were discovered in the wheat genome database. Phylogenetic trees based on all 53 genes revealed that *CBF* genes were more diverse than *ICE* and *COR* genes. Twenty-two of the 53 genes appeared to include 11 duplicated pairs. Twenty rice (*Oryza sativa*) genes and 21 sorghum (*Sorghum bicolor*) and maize (*Zea mays*) genes showed collinearity with the wheat *ICE*, *CBF* and *COR* genes. Transcriptome data and qRT-PCR analyses revealed tissue-specific expression patterns of the *ICE*, *CBF* and *COR* genes, and identified similarities in the expression pattern of genes from the same family when subjected to drought, heat, drought plus heat, and cold stress. These results provide information for better understanding the biological roles of *ICE*, *CBF*, *COR* genes in wheat.

## Introduction

Low-temperature damage in crops is a global problem for production and security with global economic losses estimated to be as high as hundreds of millions of dollars annually. Low temperatures throughout the winter are a frequent occurrence in the winter wheat (*Triticum aestivum*) production regions of northern China (Li et al., 2013). Therefore, studies have focused on elucidating the various components and underlying molecular mechanisms of the cold responsive network in plants.

The *ICE-CBF-COR* pathway is a universal pathway related to cold stress tolerance in crop species (Wang et al., 2017a; Jin et al., 2018). *ICE* (inducer of *CBF* expression) genes are members of the MYC family of transcription factors, and MYC is a subfamily of bHLH. The main structural feature of *ICE* is that it shares highly conserved regions in the bHLH domain and their C-terminal regions (Chinnusamy et al., 2003; Badawi et al., 2008; Peng et al., 2014; Lee, Henderson & Zhu, 2015; Lu et al., 2017; Jin et al., 2018). ICE factors are positive regulators of *CBF* expression in the upstream region of the low-temperature signaling pathway. There are two reported *ICE* homologs in wheat: *TaICE41* (accession no. EU562183) and *TaICE87* (accession no. EU562184). *TaICE41* and *TaICE87* bind to the *MYC* element in the promoter region of *CBF* genes such as *TaCBF IVd-B9* (Badawi et al., 2008).

*CBF* (C-repeat binding factor)/*DREB* transcription factors which belong to the *AP2/ERF* multi-gene family, are key regulators of plant freezing tolerance. A feature that distinguishes the CBF proteins from the other AP2/ERF proteins is the presence of “signature sequences”, PKKP/RAGRxKFxETRHP (PKKPAGR) and DSAWR, which are located immediately upstream and downstream, respectively, of the AP2/ERF DNA-binding domain (Skinner et al., 2005; Canella et al., 2010; Park et al., 2015; Jia et al., 2016). CBF transcription factors are absisic acid (ABA)-independent proteins involved in stress signal transduction (Shi, Ding & Yang, 2018) and essential for cold acclimation and freeze tolerance in plants (Shi et al., 2012; An et al., 2016). In wheat most of the reported cold stress-related *CBF* are located on the long arms of homoeologous group 5 chromosomes and some of them show tight linkage with the cold-resistant locus *Fr-2* (Vágújfalvi et al., 2005; Badawi et al., 2007; Knox et al., 2008; Campoli et al., 2009; Sutton et al., 2009). Cold tolerance assays showed a significant decrease in rate of survival of plants at low temperatures after the *BdCBF3* gene was knocked out in an RNAi*CBF3* mutant (Hao et al., 2017). In addition, overexpression of *MeCBF1* in seedlings of transgenic *Arabidopsis* and cassava (*Manihot esculenta* Crantz) enhanced their cold tolerance (An et al., 2017).

*CORs* (cold-responsive) generally refer to the proteins encoded by cold-responsive or cold-regulated genes, including enzymes involved in respiration and in the metabolism of carbohydrates, lipids, phenylpropanoids, and antioxidants, late embryogenesis abundant protein (LEA), stress responsive protein (SRP), cold induced (KIN), and low temperature induced (LTI) (Uemura et al., 1996; Lee et al., 1999; Guan et al., 2013). The promoters of the *COR* genes contain a cis-acting dehydration responsive elements (DRE) or C-repeats (CRT) motif (5′-CCGAC- 3′), that may be activated by members of either the CBF or DREB2 families of transcription factors (TFs) (Knight et al., 2004). Wheat COR proteins function in both ABA-independent and ABA-dependent pathways (Sun et al., 2009). Most COR proteins are induced only by low temperatures and are not sensitive to ABA, thus belonging to the ABA-independent pathway. Based on subcellular localization and function COR proteins can also be categorized into: cold-responsive chloroplast proteins; proteins involving changes in cellular; physiological and structural characteristics; proteins in both the kinase and calcium signal transduction pathways and proteins that are transmembrane receptors in wheat (Takumi et al., 2003; Ridha Farajalla & Gulick, 2007; Zhang et al., 2011). There are relatively fewer *COR* genes in ABA-dependent pathways and these act downstream from the ABA-dependent low-temperature signaling pathway where they play significant roles in protecting cells and regulating cell metabolism (Kosová, Vítámvás & Prášil, 2007). Most ABA-dependent COR proteins are LEA proteins and are responsive to cold and drought in addition to ABA (Ohno, Takumi & Nakamura, 2003; Kobayashi et al., 2004).

Due to a lack of systematic research on low temperature response signaling pathways and gene regulatory networks, cold stress signal transduction and the underlying molecular mechanisms of gene regulation are poorly understood in wheat. Although the wheat *ICE*, *CBF* and *COR* genes have been studied in a preliminary way, an ability to undertake further studies at the whole-genome level was limited by incomplete genomic information. In the present study, the most up-to-date genomic information available for wheat was used in a comprehensive survey of the three gene families. We also investigated stress responsive and differential tissue-specific expression profiles of the *ICE*, *CBF* and *COR* genes under cold stress conditions. The results allowed us to propose a cold response and regulatory mechanism for wheat.

## Material and Methods

### Identification and chromosome localization of cold defense genes

We searched for *CBF* genes in different crop species using the following methods. Firstly a Hidden Markov Model (HMM) was established using 10 CBF protein sequences from rice (*Oryza* sativa) and 17 CBF protein sequences from barley (*Hordeum vulgare*) for sequence alignment against the protein sequences of other crop genomes (Badawi et al., 2007; Skinner et al., 2005). The selection criteria of HMM was *E* < 1 × 10^−5^, with a sequence similarity of ≥50%. Secondly proteins selected by HMM but without the CBF canonical PKKR/PAGR and DSAWR motifs, as well as an AP2 domain were excluded. The remaining candidates were the predicted CBF proteins. Similarly, the COR and ICE protein sequences were established with the HMM model using the following methods. Firstly, by searching for the term “COR/ ICE wheat” in the NCBI, the corresponding genes reported in wheat was screened. Secondly, based on the currently known protein sequences of the *COR*/ *ICE* genes of barley and rice, the HMM model was constructed. By searching with this model, the wheat COR and ICE protein was obtained again (Jin et al., 2018). After repetitive sequences were removed, COR and ICE protein sequences were predicted among the remaining sequences. The predicted sequences were then validated against the NCBI-CDD (http://www.ncbi.nlm.nih.gov/Structure/cdd/wrpsb.cgi) and SMART (http://smart.embl-heidelberg.de) databases. All protein sequences without CBF, ICE and COR conserved domains were excluded.

We searched the coding sequences (CDS) of the predicted proteins using protein accession numbers, and predicted their isoelectric points and molecular masses using the online tool ExPASy (http://www.expasy.org/). To identify previously validated genes we aligned the CDS to the NCBI-EST database. CELLO (http://cello.life.nctu.edu.tw/) was used for prediction of subcellular localization of the genes. Sequence and chromosome information were obtained from the Ensembl Plant Database (http://plants.ensembl.org/Triticum_aestivum/Info/Index), including DNA, CDS and protein sequences for wheat, and protein sequences from other species.

### Phylogenetic analysis

We combined our cold defense protein sequences from wheat and other model plants and conducted sequence alignment using T-COFFEE. We used IQTREE (JTT model) and PhylML (JTT model) to develop maximum likelihood (ML) phylogenetic trees. Bootstrap replications were set as 1,000, and other parameters were set as default. The phylogenetic trees were prepared using the online tool Evolview (http://www.evolgenius.info/evolview/).

### Gene structures, conserved motifs and cis-acting elements

We submitted CDS and full-length DNA sequences of the predicted wheat *ICE*, *CBF* and *COR* genes to the GSDS 2.0 (Gene Structure Display Server) for prediction of gene structure. The corresponding derived protein sequences were then submitted to MEME (http://meme-suite.org/index.html) for motif analysis. The maximum motif number was set as 5, and the motif lengths were set from 6 to 200 aa. The online tool Evolview (http://www.evolgenius.info/evolview/) was used for gene structure analysis and motif identification. The 2,000 bp sequences upstream of the promoters of the *ICE*, *CBF* and *COR* genes were submitted to the New PLACE Database for analysis of cis-acting elements (https://www.dna.affrc.go.jp/PLACE/).

### Duplication and co-linear analyses of *ICE*, *CBF* and *COR* genes

We identified duplicate genes using the following criteria: (a) alignment covered >80% of the longer gene; (b) the aligned region had >80%identity and (c) only one duplication event was accepted for tightly linked genes (Voet et al., 2013). We conducted visualized analysis for the gene duplications and chromosome localizations using Circos (Krzywinski, Schein & Birol, 2009). The Multiple Collinearity Scan toolkit (MCScanX) with default parameters was adopted to analyze gene collinearity between wheat and other selected species (rice, sorghum and maize) (Wang et al., 2012).

### Expression profiling of the *ICE, CBF* and *COR* genes

RNA-seq data from different tissues (roots, stems, leaves, spikes, and grain) (E-MTAB-4484) from wheat plants under different stress conditions (accession numbers GSE58805 for cold stress and SRP045409 for drought, heat and drought plus heat stress) were downloaded from high-throughput data provided by EMBL and NCBI-SRA and used to analyze the expression patterns of the predicted *ICE*, *CBF* and *COR* genes from wheat. The results were visualized using MeV 4.9.0.

### Plant materials and experimental design for qRT-PCR

To validate the reliability of RNA-seq results, we measured the transcription levels of differentially expressed genes using qRT-PCR (Livak & Schmittgen, 2001). Gene sequences were downloaded from IWGSC, and primers were designed using Primer 3 (http://frodo.wi.mit.edu/) (Table S1). Samples were run on an ABI7900 system in 20 µl reactions. Relative expression levels of genes were calculated by the 2^−ΔΔ*CT*^ method. Statistical analysis was calculated using SPSS 21.0 (IBM, Armonk, NY, USA).

Wheat cultivar Chinese Spring (CS) was used for qRT-PCR. Seeds were sterilized using 0.5% NaClO, washed in ddH_2_O, and immersed in water for 24 h at room temperature. The germinated seedlings were transplanted into pots (diameter: 30 cm, height: 40 cm, 5 per pot). Experiments were performed in 3 replications, and each replicate had 3 technical replicates in 3 pots. Vernalized seedlings were moved to a growth chamber (light/darkness: 16/8 h; 22/18 °C). Root tissues were sampled at the three-leaf stage; leaf tissues were sampled at cotyledon emergence; stem tissues at initiation of stem elongation; spike tissues at the two nodes or internodes visible stage while grains were collected at the ripening stage. Following the steps above, simultaneously germinated seeds were placed in growth boxes (25 seeds per box). There were three replications of control and stress-treated groups, and each replicate consisted of 3 boxes. All boxes were placed in a growth chamber set as indicated above. Seedlings at the one-leaf stage were placed in one-quarter Hoagland’s solution, and stress treatments were applied at the three-leaf stage. Procedures for stress treatment were as follows: 1. drought stress: Hoagland’s solution with 20% PEG-6000 (−1.23 MPa) used to treat the seedlings; 2. heat stress: seedlings were moved to a 40 °C growth chamber; 3. drought plus heat: seedlings were moved to a 40 °C growth chamber and treated with Hoagland’s solution +20% PEG-6000 (−1.23 MPa); and 4. low temperature stress: seedlings were moved to a 4 °C growth chamber. Samples were collected 1 h and 6 h after the beginning the stress treatment. Seedlings from the control group (22/18 °C, one-quarter Hoagland’s solution) were sampled at the same time. Samples were immediately frozen in liquid nitrogen and stored at −80 °C. Each treatment had 3 biological replicates.

## Results

### Identification *ICE*, *CBF* and *COR* genes in different species

Alignment results from 42 species indicated that the numbers of *ICE*, *CBF* and *COR* genes were 274, 131 and 188, respectively. Numbers per species ranged from 1 to 53. Wheat had the greatest number of genes with 53 (Table S2), including 5 *ICE*, 37 *CBF* and 11 *COR* genes (Table S3). No cold tolerance-related genes were identified in species *Chlamydomonas reinhardtii*, *Coccomyxa subellipsoidea*, *Micromonas pusilla*, *Ostreococcus lucimarinus*, and *Volvox carteri.*

### Evolutionary relationships among *ICE*, *CBF* and *COR* genes

Phylogenetic trees were established for the 593 *ICE*, *CBF* and *COR* genes from 37 species. Using bootstrap computing techniques, these genes were categorized into *ICE*, *CBF* and *COR* gene families (Fig. S1) with *CBF* being the largest family.

**Figure 1:**
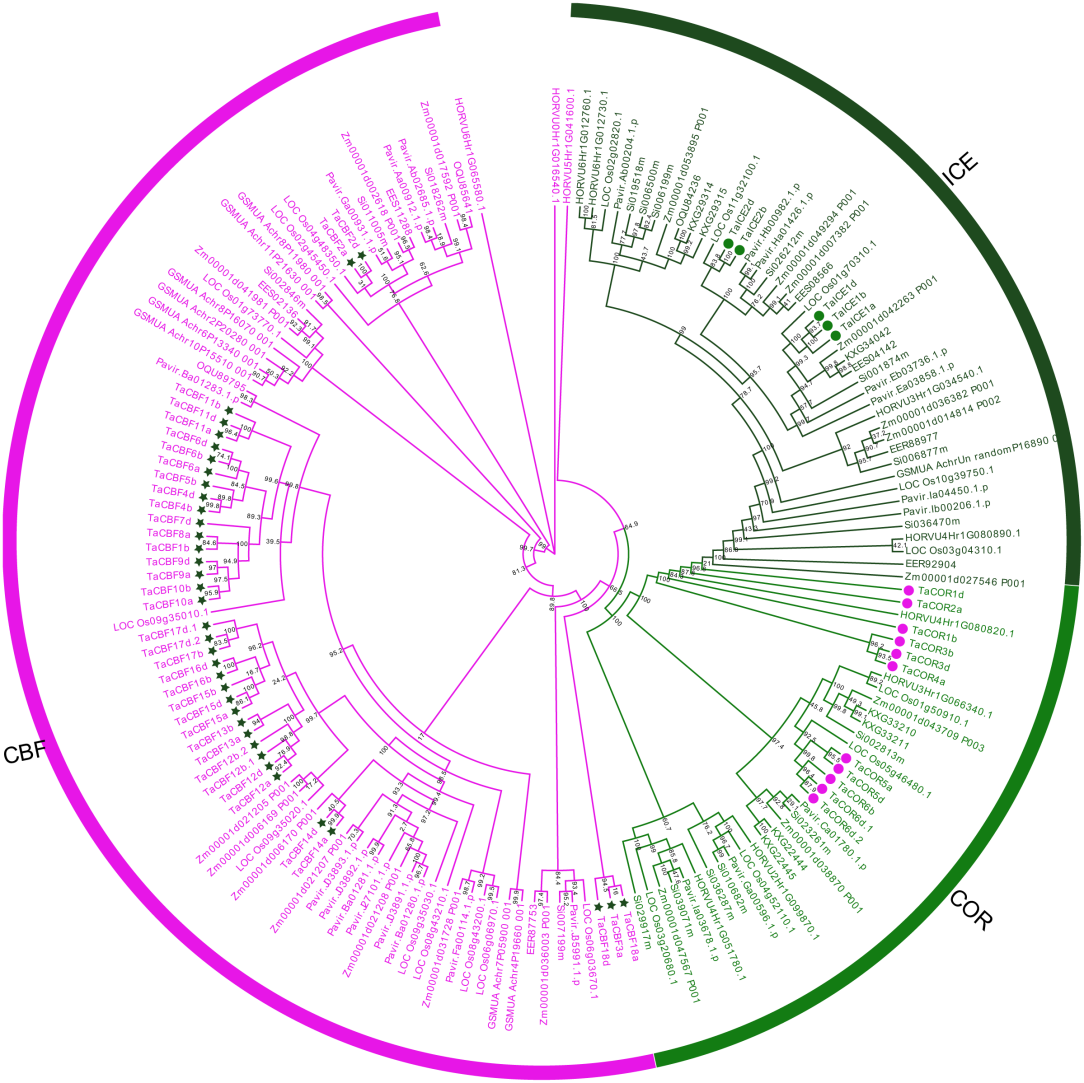
Phylogenetic trees established for *ICE*, *CBF* and *COR* gene families in monocots. *ICE*, *CBF* and *COR*, are represented by the dark green, magenta and green, respectively. *ICE*, *CBF* and *COR* genes in wheat are marked with symbols.

Based on differences in conserved domains within each ICE, CBF and COR protein family, as well as their evolutionary relationships, we established the phylogenetic trees for these proteins from monocots using maximum likelihood (ML). *ICE* and *COR* had a closer evolutionary relationship than either had with CBF (Fig. 1). Phylogenetic trees also revealed the phylogenetic relationships among the 53 ICE, CBF and COR proteins in wheat and those in other species, e.g., rice, maize and barley (Fig. 1).

### Motif and gene structure analysis of proteins encoded by *ICE*, *CBF* and *COR* genes

**Figure 2:**
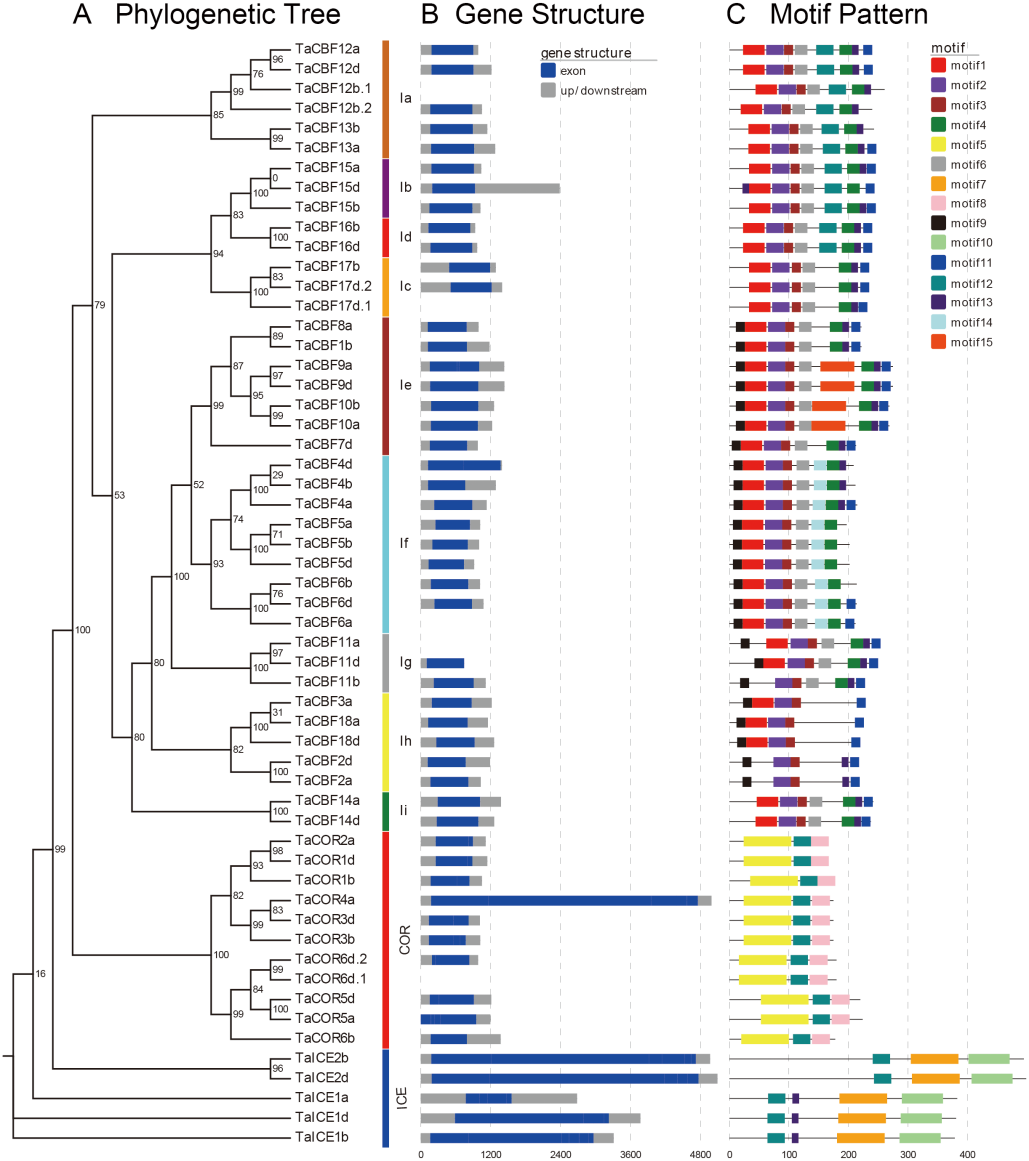
Phylogenetic relationships, gene structure and architecture of conserved protein motifs in the *ICE*, *CBF* and *COR* genes of wheat. A. Evolutionary relationships (ML phylogenetic trees) among the *ICE*, *CBF* and *COR* genes. Clusters are shown in different colors; B. Gene structure diagrams of wheat *ICE*, *CBF* and *COR* genes. Gray parts indicate 5′ and 3′ UTR; blue parts indicate exons. C. Motif structure diagrams of wheat *ICE*, *CBF* and *COR* genes. The motifs, numbers 1–15, are displayed in different colored boxes. Sequence information for each motif is provided in Fig. S2.

Motif analyses of proteins encoded by the predicted 53 cold tolerance-related genes in wheat showed the presence of different conserved motifs in subfamilies of *ICE*, *CBF* and *COR*. For example, motifs 2 and 3 were present in all *CBF* genes, while motif 14 was present only in the Ig subgroup. Motifs 7 and 10 were present only in the *ICE* genes, whereas motifs 5 and 8 were present only in *COR* genes (Fig. 2C, Fig. S2).

Gene structure analysis revealed that *ICE*, *CBF* and *COR* genes had no introns except for two *CBF* genes and one *COR* gene. *TaCBF11a* and *TaCBF12b.1* had 1 intron each and *TaCOR6d.1* had 2 introns (Fig. 2B, Fig. S3).

### Analysis of cis-acting elements

A total 153 cis-acting elements related to cold tolerance were identified in wheat, including those responsive to plant hormones and abiotic stresses, and others related to pollen and endosperm development. For example, the element MYCCONSENSUSAT (CANNTG) is responsive to cold and drought; GATABOX (GATA), IBOXCORE (GATAA), and GT1CONSENSUS (GRWAAW) are responsive to light; GTGANTG10 (GTGA) and POLLEN1LELAT52 (AGAAA) are related to pollen development, while DOFCOREZM (AAAG) is related to endosperm development (Tables S4 and S5).

### Localization and co-linear analysis of *ICE*, *CBF* and *COR* genes

**Figure 3:**
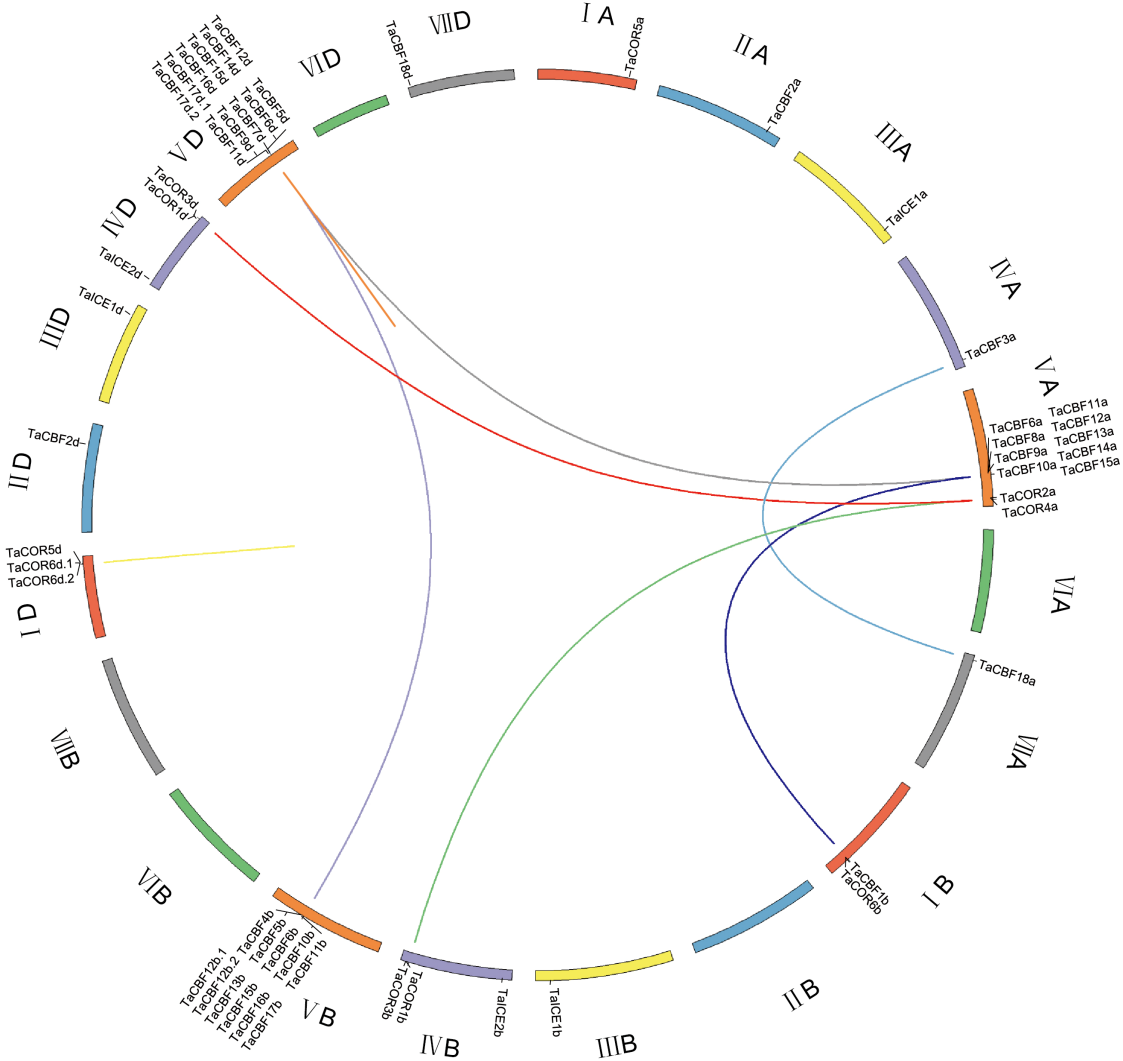
Chromosome location and duplicated wheat *ICE*, *CBF* and *COR* genes. The seven homoeologous chromosome groups are displayed in different colors. Duplicated gene pairs are linked by lines and the different color lines to represent the duplicated relationship.

*ICE*, *CBF* and/or *COR* genes were located on all chromosomes except 2B, 6A, 6B, 6D and 7B. *CBF* genes were mainly located on chromosomes 5A, 5B and 5D; all 11 genes on each of chromosomes 5B and 5D were *CBF* genes. *COR* genes were located on chromosomes 1A, 1B, 1D, 4B and 4D. *ICE* genes were located on chromosomes 3A, 3B, 3D, 4B and 4D (Fig. 3). Gene duplication analysis of *ICE*, *CBF* and *COR* genes detected 11 pairs of duplicates accounting for 22 genes (Fig. 3, Table S6).

**Figure 4:**
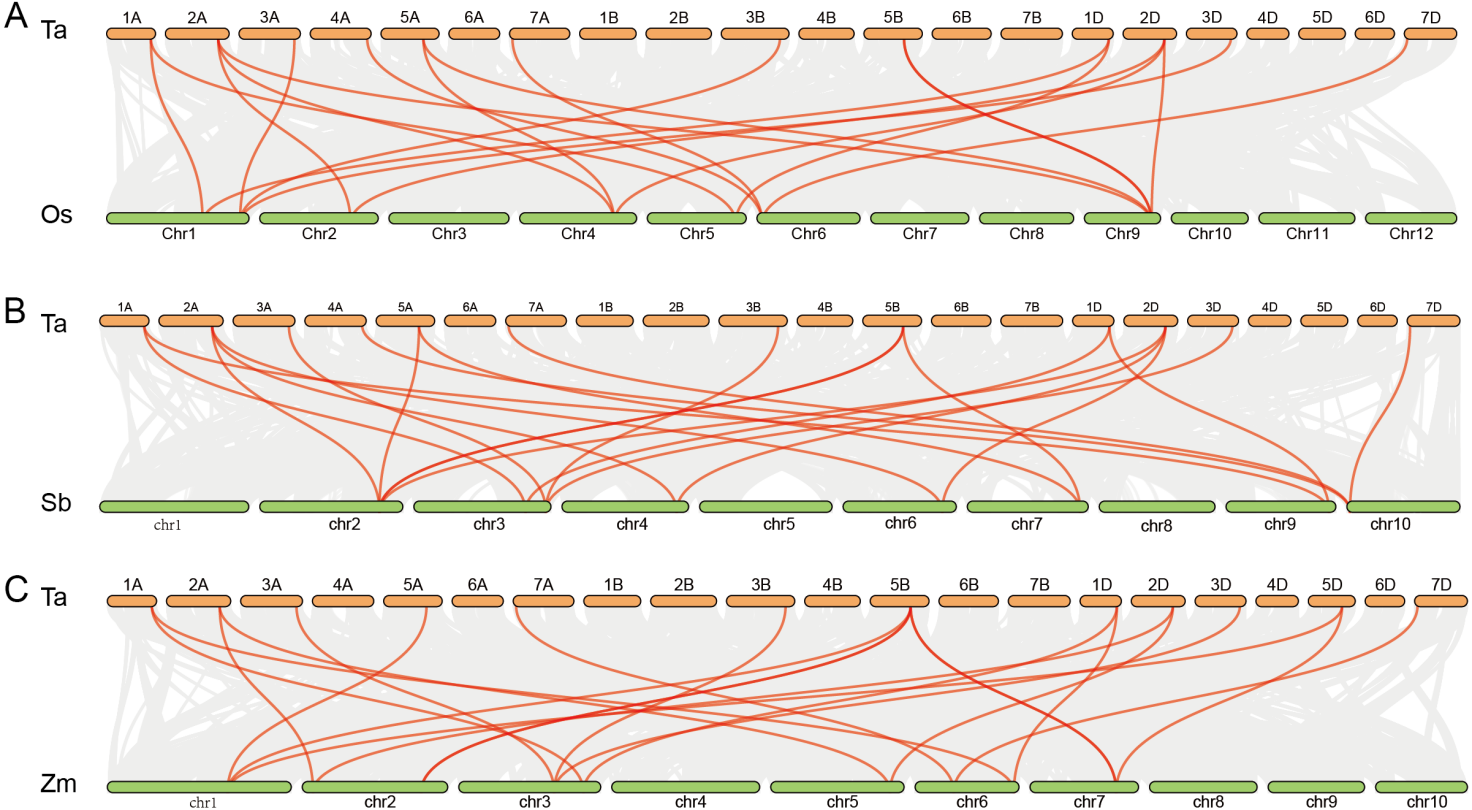
Co-linear analysis of wheat *ICE*, *CBF* and *COR* genes with rice, sorghum and maize genes. Gray lines in the background indicate the collinear blocks within wheat and other plant genomes; red lines highlight syntenic *ICE*, *CBF* and *COR* gene pairs. The species names with the prefixes ‘Ta’, ‘Os’, ‘Sb’, and ‘Zm’, are for common wheat, rice (A), sorghum (B) and maize (C), respectively.

Further analysis showed that twenty rice genes were co-linear with the cold tolerance-related wheat genes including 13 *CBF* genes, 3 *ICE* genes and 4 *COR* genes. Twenty-one sorghum and maize genes had co-linear relationships with cold tolerance-related genes in wheat, including 14 *CBF* genes, 3 *ICE* genes and 4 *COR* genes. Among these, *TaCBF2a*, *TaCBF2d*, *TaCBF10b*, *TaCBF18a*, *TaCBF18d*, *TaICE1a*, *TaICE1b*, *TaICE1b*, *TaCOR5a* and *TaCOR5d* had co-linear relationships with genes in all three species (rice, sorghum and maize) (Fig. 4, Table S7).

### Expression profiling of wheat *ICE*, *CBF* and *COR* genes with RNA-seq

**Figure 5:**
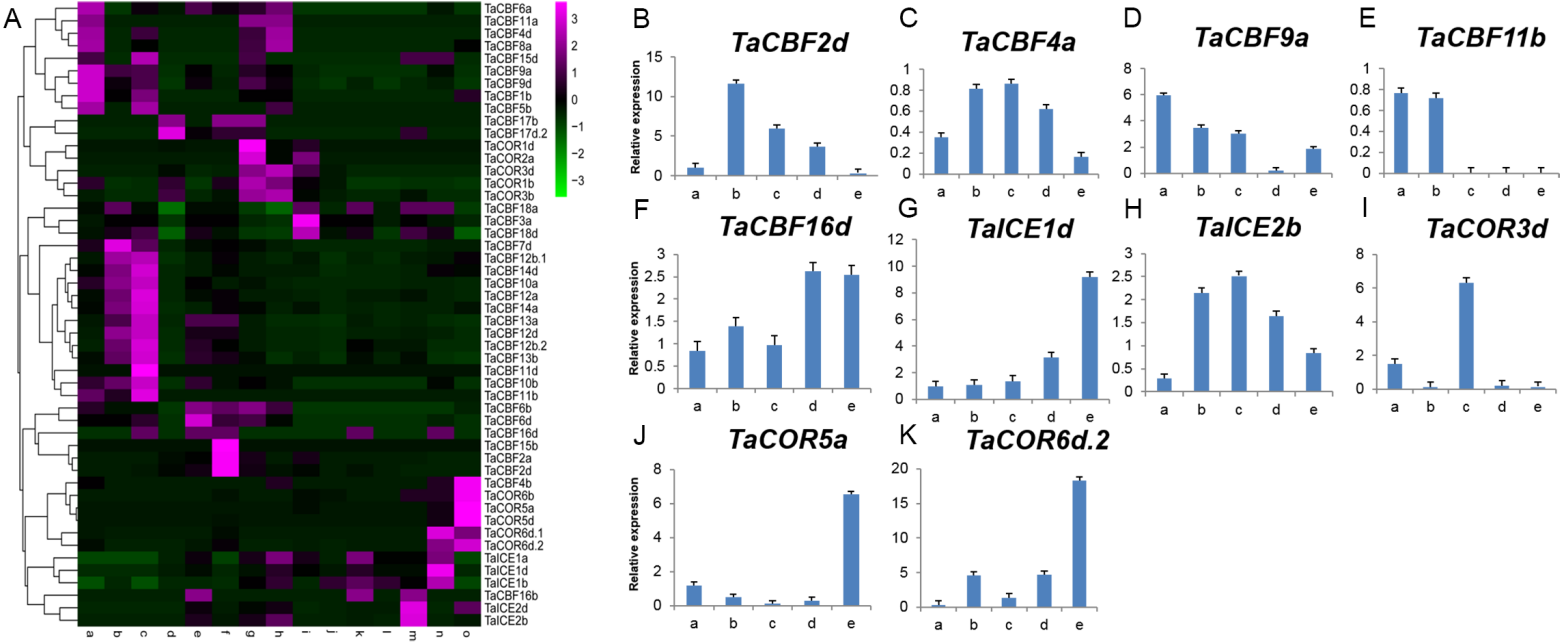
Expression profiling of *ICE*, *CBF* and *COR* genes in different tissues of wheat. A. Cluster heat-map analysis of *ICE*, *CBF* and *COR* genes in different tissues and during different developmental stages: root tissue at: a. cotyledon emergence; b. maximum stem length; c. three leaves visible; stem tissue at: d. 50% of flowers open; e. initiation of stem elongation; f. two nodes or internodes visible; leaves at: g. main shoot and axillary shoots visible at three nodes; h. cotyledon emergence; i. mid grain-fill; spike: j. mid-anthesis; k. two nodes or internodes visible; l. maximum stem length; grain : m. mid grain-fill; n. whole plant grain formation stage 70% to final size; o. grain ripening stage. B–K. Expression levels of two *ICE*, five *CBF* and three *COR* genes in five tissues validated by qRT-PCR: root tissue at: a. three leaves visible; stem tissue at: b. initiation of stem elongation; leaf tissue at: c. cotyledon emergence; spike: d. two nodes or internodes visible ; grain: e. whole plant grain ripening stage.

Expression profiling using RNA-seq data for *ICE*, *CBF* and *COR* genes expressed during different developmental stages in different wheat tissues revealed the specificity of expression patterns (Fig. 5A, Table S8). For example, *TaCBF15b*, *TaCBF2a* and *TaCBF4b* were up-regulated mainly at stage f (two nodes or stem internode visible), whereas *TaCBF4b*, *TaCOR6b*, *TaCOR5a* and *TaCOR5d* were up-regulated mainly in stage o (grain ripening stage). To validate the reliability of the RNA-seq data, we analyzed the expression of two *ICE*, five *CBF* and three *COR* genes in five tissues (roots, stems, leaves, spikes, and grain) by qRT-PCR. *TaCBF4a*, *TaCBF16d*, *TaICE1d* and *TaICE2b* were expressed in all five tissues. The expression levels of *TaICE1d* increased with the progression of growth stages (Figs. 5B–5K).

### Expression profiling of *ICE*, *CBF* and *COR* genes in response to abiotic stress

**Figure 6:**
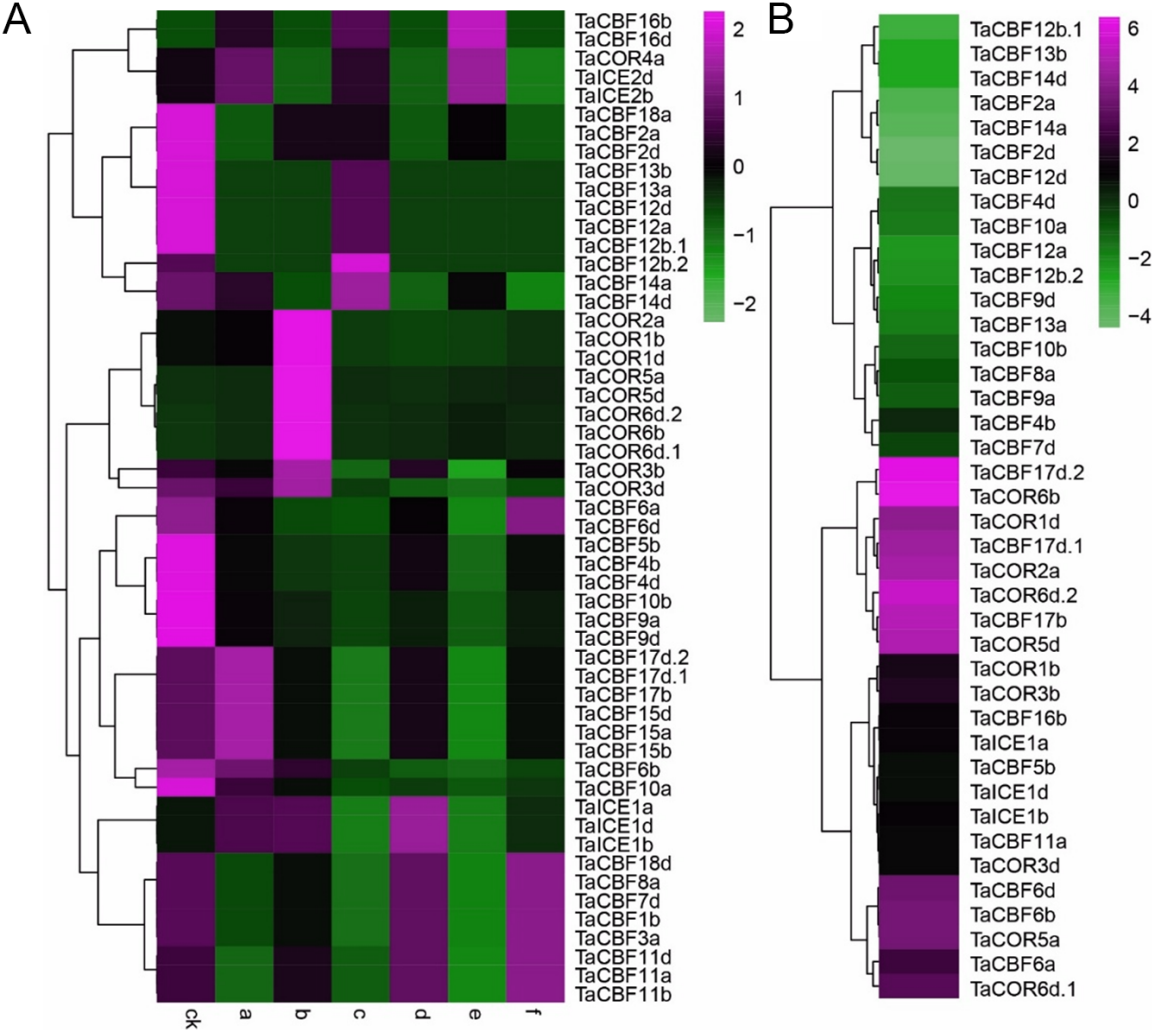
Expression profiling of *ICE*, *CBF* and *COR* genes in wheat under different abiotic stresses. A. Heat-map analysis of *ICE*, *CBF* and *COR* genes under drought, heat and drought plus heat stresses. CK, control group; a. drought treatment for 1 h; b. drought treatment for 6 h; c. heat treatment for 1 h; d. heat treatment for 6 h; e. drought + heat treatment for 1 h; f. drought + heat treatment for 6 h. B. Changes in expression pattern of *ICE*, *CBF* and *COR* genes after two weeks of cold stress.

Expression profiling of the 53 *ICE*, *CBF* and *COR* genes under different abiotic stress conditions showed that expression was induced by 1 h of heat or drought (Fig. 6A). In addition, the results showed that genes from the same family had similar expression patterns. For example, the expression patterns of *TaCBF11a*, *TaCBF11b* and *TaCBF11d* were almost identical under the same stress conditions, and expression patterns of 10 *COR* genes were similar (Fig. 6A, Table S8). In contrast, expression patterns of some other genes were significantly different under cold stress, especially genes from the *COR* and *ICE* families that were up-regulated following exposure to low temperature (Fig. 6B).

**Figure 7:**
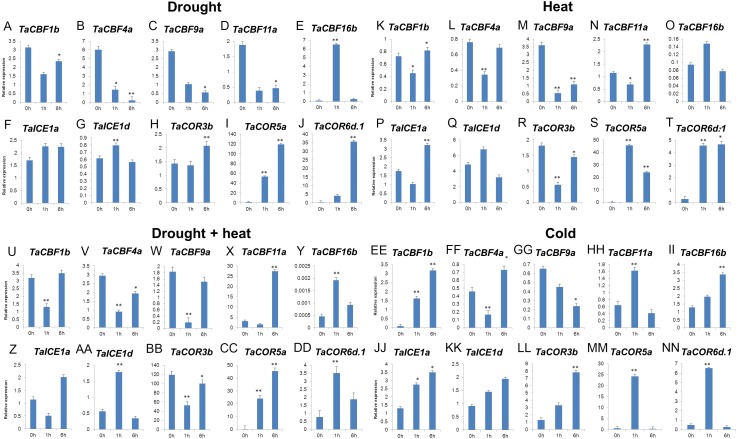
Expression levels of 2 *ICE*, 5 *CBF* and 3 *COR* genes in wheat under drought (A–J), heat (K–T), drought + heat (U-DD), and cold (EE-NN) stresses validated by qRT-PCR.

Validation of 2 *ICE*, 5 *CBF* and 3 *COR* genes under different stress conditions by qRT-PCR revealed that 6 genes (*TaCBF11a*, *TaCBF16b*, *TaICE1a*, *TaICE1d*, *TaCOR5a* and *TaCOR6d.1*) were induced by all four treatments: drought, heat, drought plus heat, and cold treatments. Three genes, *TaCBF1b*, *TaCBF4a*, *TaCOR3b* were induced by cold treatment only while only one gene (*TaCBF9a*) was inhibited by cold stress treatment (Fig. 7).

## Discussion

### Evolution and expansion of *ICE*, *CBF* and *COR* genes

Cold tolerance in crops is a complex quantitative trait controlled by polygenes. This type of trait is induced under specific conditions, and only co-expression of multiple genes can enhance cold tolerance in crops. *ICE*, *CBF* and *COR* are cold tolerance-responding functional and regulatory genes that participate directly or indirectly in defense against low temperature (Wang et al., 2017a).

In the present study, the total number of *ICE*, *CBF* and *COR* genes detected in 37 species ranged from 1 to 53 (Table S2). The numbers of these genes had a history of increasing from 0 in the ancient *Chlorophyta* species to an average of 4.5 in mosses, and an average of 21.37 in monocots (Table S2).

Although diversification of dicots from monocots occurred about 140–150 million years ago the number of *ICE*, *CBF* and *COR* genes in *Arabidopsis* is almost identical to that in cereal crops, indicating a similar rate of increase in numbers in both dicots and monocots (Table S2) (Wendel et al., 2016). Diversification of cereal crops occurred 50–70 million years ago (Strömberg, 2011), and the size of the *ICE*, *CBF* and *COR* gene families were similar among cereal crop species, except for wheat. The number of *ICE*, *CBF* and *COR* genes in wheat is almost 2.5 times of that in maize, rice and sorghum. The most likely cause of this difference is hexaploidization of the wheat genome (Table S2) (Zimin et al., 2017; Wang et al., 2017b). However, the wheat genome might have gone through an expansion in numbers of *ICE*, *CBF* and *COR* genes from the time that the crop was introduced to high-latitude areas from low-latitude areas.

### Expression profiling of *ICE*, *CBF* and *COR* genes in wheat

Growth, development and stress responses in plants are closely related to gene expression and regulation. Therefore, studies on expression patterns of *ICE*, *CBF* and *COR* genes in wheat are important for understanding their underlying mechanisms (Chung et al., 2016; Chae et al., 2017). Investigation of expression patterns of *ICE*, *CBF* and *COR* genes in wheat roots, stems, leaves, spikes and grains using RNA-seq data and qRT-PCR revealed significantly different expression levels among the 53 *ICE*, *CBF* and *COR* genes across tissues. *ICE* genes were mainly up-regulated in grains, *CBF* in roots and stems, and *COR* in leaves and grains. (Fig. 5A). Previous studies revealed significant differences in *CBF* gene expression levels between leaf and tree bark tissues in peach (*Prunus persica* L.) (Artlip et al., 2013). Gene expression was largely down-regulated in leaf tissues at 4 °C, but was slightly up-regulated in tree bark tissues. qRT-PCR analysis of 2 *ICE*, 5 *CBF* and 3 *COR* genes in 5 tissues (roots, stems, leaves, spikes, and grain) revealed that *TaCBF4a*, *TaCBF16d*, *TaICE1d* and *TaICE2b* were expressed in all tissues, but the highest expression levels of *TaICE2b* were in stem and leaf tissues (Figs. 5B–5K). Previous studies revealed that in *Vitis amurensis*, the *ICE1* gene was expressed in root, stem, leaf and petiole tissues after cold stress treatment. Expression levels were the highest in root tissues at the beginning of cold stress, but an increase was observed in stem and leaf tissues in the later period of cold stress (Dong et al., 2013). *ICE1* in radish (*Raphanus sativus* L.) seedlings showed relatively high expression levels in root and stem tissues (Man et al., 2017). Therefore, *ICE*, *CBF* and *COR* genes have tissue-specific expression patterns that vary from species to species.

*ICE*, *CBF* and *COR* genes have a vital role in stress tolerance and acclimatization in plants (Oh et al., 2007; Ganeshan et al., 2008; Chen et al., 2012; Soltész et al., 2013; Dou et al., 2015). Transformation of *HvCBF4* into rice enhanced the tolerance of plants to drought, salinity and low temperatures (Oh et al., 2007). Transgenic barley lines with the *TaCBF14* and *TaCBF15* genes also had enhanced cold tolerance compared to wild-type plants (Soltész et al., 2013). Heat stress treatment of transgenic potatoes (*Solanum tuberosum*) significantly induced the *AtCBF3* gene when the temperature was 40 °C or higher (Dou et al., 2015). Ganeshan et al. (2008) analyzed the expression patterns of *COR* genes in wheat leaf and crown tissues using near-isogenic lines and found that the transcription levels of *COR* genes reached their maximum at 2 days after cold stress treatment. Previous studies showed that expression of *CdICE1* in *Chrysanthemum grandiflorum* enhanced its tolerance of cold, drought and salt stresses (Chen et al., 2012). In our study, qRT-PCR validation of 10 genes under different stresses showed that *TaCBF11a*, *TaCBF16b*, *TaCOR5a*, *TaCOR6d.1*, *TaICE1a* and *TaICE1d* were induced simultaneously under drought, heat, drought plus heat, and cold stresses (Fig. 7), indicating that the predicted *CBF*, *COR* and *ICE* genes were responsive to abiotic stresses.

Our study also showed similarities in the expression patterns of genes from the same family (Fig. 6A, Table S8). The *ICE*, *CBF* and *COR* genes each have a typical conserved motif (Fig. 2C) that functions similarly in transcriptional regulation, thereby presenting similar transcript profiles upon stress. Dubos et al. (2010) reported that different family members of the MYB transcription factor family are involved in regulation of the same metabolic pathway. Furthermore, genes in the same family normally exhibit similar expression profiles in different plant tissues. According to the present study, the TaCOR6b, TaCOR5a, TaCOR2d, TaCOR6d.1 and TaCOR6d.2 proteins share the same conserved motif and are expressed similarly in different tissues (Fig. 2C). Six *PmCBF* genes from *Prunus mume* shared similar expression patterns under cold stress (Zhao et al., 2018). *PmCBF* expression was high in stems but only moderate in flower buds, leaf buds, and leaves, and low in flowers, fruit, and seed.

## Conclusions

Five *ICE*, 37 *CBF* and 11 *COR* genes were characterized and they had high similar exon-intron structures and motif compositions within the same family. Phylogenetic comparison from monocots showed *ICE* and *COR* had a closer evolutionary relationship than either had with *CBF*. Furthermore, the expression profiles of wheat *ICE*, *CBF* and *COR* genes provided the candidates for further functional characterization of *ICE*, *CBF* and *COR* genes with an aim of wheat improvement. In conclusion, this study contributed to better understanding the roles of *ICE*, *CBF* and *COR* genes involving in growth and development as well as in response to stresses in wheat.

##  Supplemental Information

10.7717/peerj.8190/supp-1 Figure S1 Phylogenetic trees established for 593 *ICE*, *CBF* and *COR* genes in 37 species *ICE*, *CBF* and *COR*, are represented by the dark green, magenta and green, respectively.

10.7717/peerj.8190/supp-2 Figure S2 The consensus sequence of motif 1-15 from wheat ICE, CBF and COR proteins

10.7717/peerj.8190/supp-3 Figure S3 Gene structure diagrams of *TaCBF6a*, *TaCBF11a*, *TaCBF17d.1*, *TaCBF12b.1* and *TaCOR6d.1* genes The blue, gray and yellow parts indicate 5′ or and 3′ UTR, introns and CDS, respectively.

10.7717/peerj.8190/supp-4 Table S1 Primer sequences of 16 genes used for qRT-PCR validation

10.7717/peerj.8190/supp-5 Table S2 Distribution and numbers of *ICE*, *CBF* and *COR* genes in different species

10.7717/peerj.8190/supp-6 Table S3 Distribution and numbers of wheat *ICE*, *CBF* and *COR* genes

10.7717/peerj.8190/supp-7 Table S4 Cis-acting elements related to wheat *ICE*, *CBF* and *COR* genes

10.7717/peerj.8190/supp-8 Table S5 Function and annotation of cis-acting elements related to wheat *ICE*, *CBF* and *COR* genes

10.7717/peerj.8190/supp-9 Table S6 Duplication relationship among 11 pairs of wheat *ICE*, *CBF* and *COR* genes

10.7717/peerj.8190/supp-10 Table S7 Co-linear analysis of wheat *ICE*, *CBF* and *COR* genes with rice, sorghum and maize genes

10.7717/peerj.8190/supp-11 Table S8 RNA-seq data for 53 ICE, CBF and COR genes used in this study

10.7717/peerj.8190/supp-12 Data S1 Raw data of qRT-PCR

## Additional Information and Declarations

### Data Availability

The following information was supplied regarding data availability: The raw measurements are available in the Supplemental Files.
