# Supplementary figures and images for: Characterization and expression profiling of the ICE-CBF-COR genes in wheat
Source: PeerJ. 2019 Nov 29;7:e8190. doi: 10.7717/peerj.8190 (PMC6886486; doi:10.7717/peerj.8190)

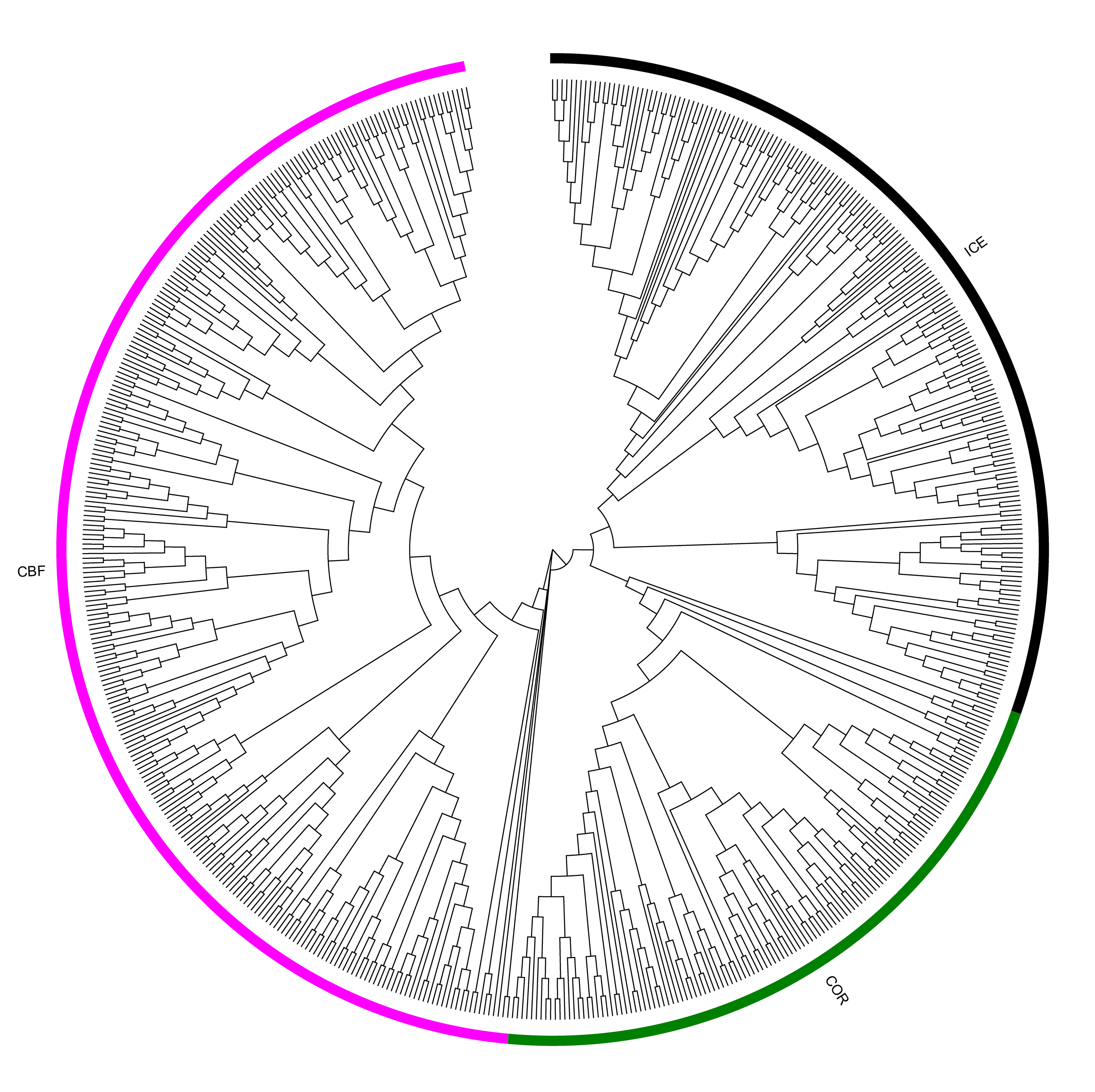

Supplement: Figure S1 — ICE, CBF and COR, are represented by the dark green, magenta and green, respectively. [file peerj-07-8190-s001.png]

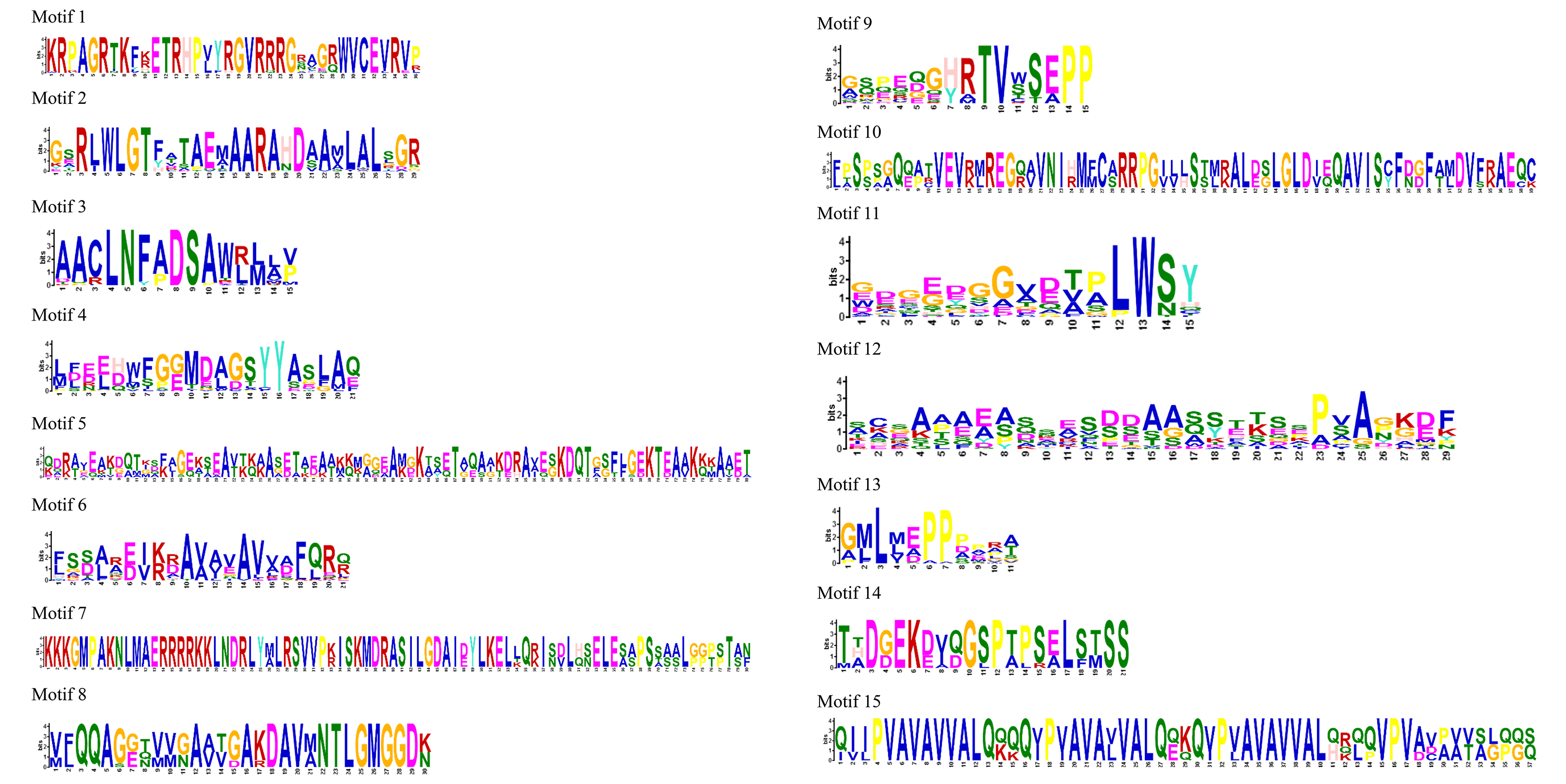

Supplement: Figure S2 [file peerj-07-8190-s002.png]

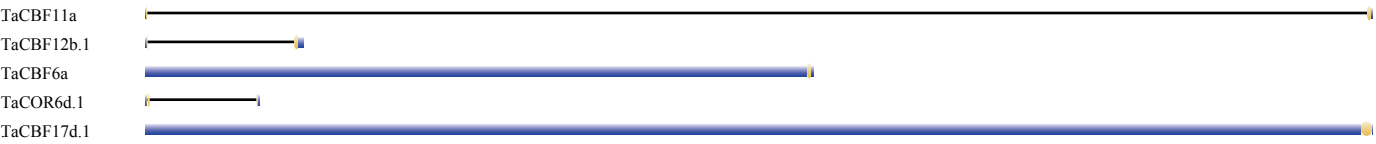

Legend:

■ CDS   ■ upstream/ downstream   — Intron

Supplement: Figure S3 — The blue, gray and yellow parts indicate 5′ or and 3′ UTR, introns and CDS, respectively. [file peerj-07-8190-s003.pdf]
